# Pseudotimecascade Visualizes Gene Expression Cascade in Pseudotime Analysis

**DOI:** 10.34133/csbj.0007

**Published:** 2026-03-06

**Authors:** Changxin Wan, Beijie Ji, Zhicheng Ji

**Affiliations:** ^1^ Department of Biostatistics and Bioinformatics, Duke University School of Medicine, Durham, NC, USA.; ^2^ Program of Computational Biology and Bioinformatics, Duke University School of Medicine, Durham, NC, USA.; ^3^ Department of Statistical Science, Duke University, Durham, NC, USA.

## Abstract

Single-cell transcriptomic technologies enable the reconstruction of dynamic biological processes such as cell development and differentiation. While existing pseudotime methods allow the analysis of temporal expression patterns, they primarily focus on individual genes, overlooking the coordinated programs that drive cellular transitions. We introduce Pseudotimecascade, a tool for visualizing and comparing multi-gene expression cascades along pseudotime. In addition, it links these cascades to biological functions by identifying stage-specific pathways. Applied to hematopoietic stem cell differentiation, Pseudotimecascade highlights regulatory hierarchies and stage-specific processes, offering a deeper understanding of gene programs that govern cell fate decisions.

## Introduction

Pseudotime analysis [1–4] and RNA velocity analysis [5,6] have become standard tools for deciphering gene expression dynamics in continuous biological processes [7–10]. These methods computationally order cells based on their transcriptomic profiles, and the resulting ordering reflects each cell’s position in cell developmental or differentiation processes. A fundamental step after obtaining the pseudotime ordering is to analyze and visualize gene expression patterns along the trajectory. This provides insights into the mechanisms by which key genes drive biological processes such as immune response, cancer progression, and neuronal differentiation [7–10].

Several computational methods have been developed to analyze gene expression patterns along pseudotime. For example, Monocle and TSCAN [1,2] introduced statistical testing procedures based on generalized additive models (GAMs) [11]. PseudotimeDE [12] further refined the statistical framework to improve differential expression testing along pseudotime. Lamian [13] is another statistical model that enables the identification of temporally differential genes across multiple samples. Many pseudotime analysis tools [1–4] also provide functions for visualizing gene expression patterns along pseudotime.

A major limitation of these methods is that the analysis of temporal expression patterns is performed on individual genes in isolation. While examining single-gene dynamics can yield useful insights, biological processes such as differentiation and development are rarely driven by single genes alone. Instead, they are orchestrated by coordinated gene programs comprising multiple genes, each contributing distinct functions at different stages of the process. Jointly analyzing the expression cascade of multiple genes enables the discovery of these coordinated programs, captures temporal dependencies among genes, and provides deeper insights into how complex cellular transitions are regulated. In addition, these methods require users to manually select and inspect genes, making it difficult to systematically identify stage-specific programs or regulatory hierarchies at scale.

**Fig. 1.**
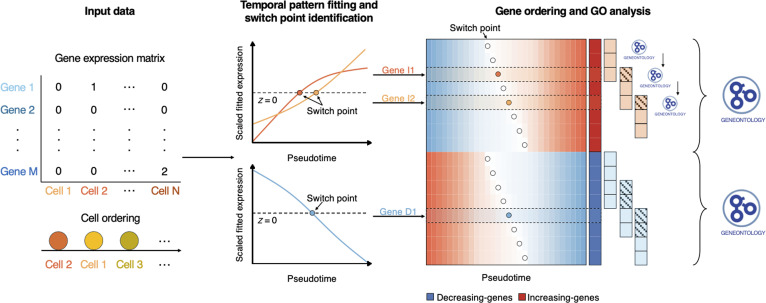
Overview of the Pseudotimecascade analysis pipeline. Starting from a gene-by-cell expression matrix, cells are first ordered along a pseudotime trajectory inferred by an external method. For each gene, scaled expression values are fitted along pseudotime, and temporal patterns are classified based on the direction of expression changes. Switch points are identified as characteristic positions along pseudotime where gene expression dynamics shift. Genes are subsequently ordered within each temporal pattern according to their switch-point locations, resulting in a pattern-aware gene ordering. Finally, functional enrichment analyses are conducted on the ordered gene sets, including pattern-level GO enrichment and temporal GO analysis to characterize functional programs along pseudotime.

To address these limitations, we developed Pseudotimecascade (Fig. 1), a comprehensive tool for visualizing and comparing temporal gene expression patterns of multiple genes in pseudotime analysis. Rather than focusing on individual genes, Pseudotimecascade jointly analyzes multiple genes to uncover their relative temporal ordering and stage-specific activation patterns. A central innovation of Pseudotimecascade is its temporal gene ontology (GO) enrichment analysis, which characterizes continuous changes in functional programs along pseudotime. Although sliding-window approaches have previously been used to study dynamic expression quantitative trait loci (eQTLs) [14] or to smooth false discovery rates in gene set enrichment tests [15], these methods are not directly applicable to pseudotime analysis in single-cell RNA-sequencing (scRNA-seq) data and do not capture continuous functional dynamics along a cellular trajectory. By integrating multi-gene expression cascades with temporal functional enrichment, Pseudotimecascade enables the identification of regulatory hierarchies and stage-specific biological processes that are not readily accessible with existing pseudotime methods. This substantially enhances biological interpretability and provides a complementary layer of insight beyond gene-level differential expression and visualization. When applied to 2 real scRNA-seq datasets, Pseudotimecascade uncovers the gene expression cascades that drive hematopoietic stem cell (HSC) differentiation. By extending pseudotime analysis from individual genes to multiple genes, Pseudotimecascade offers deeper insights into the dynamics of gene expression programs in pseudotime analysis.

## Results

**Fig. 2.**
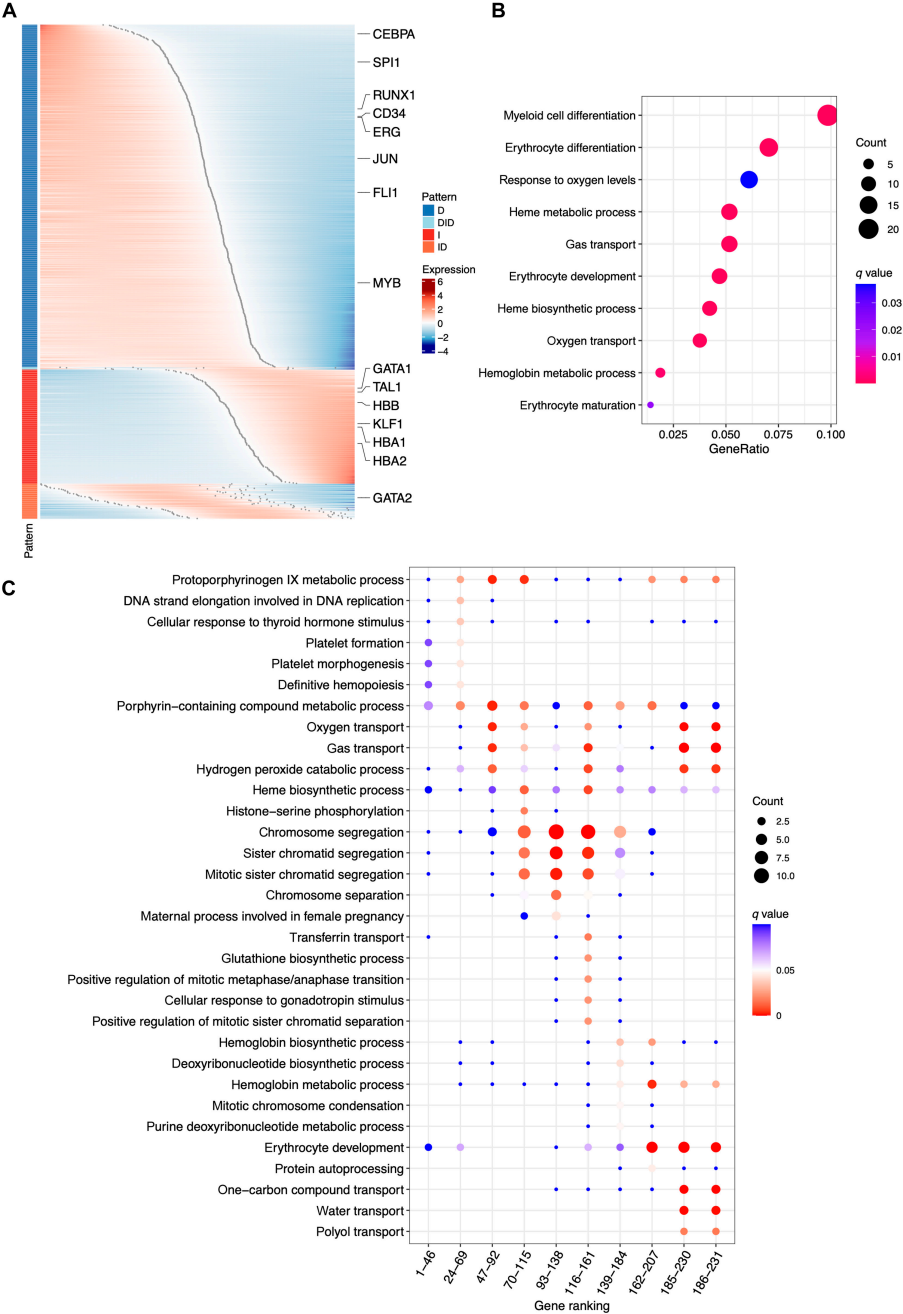
Single-sample Pseudotimecascade analysis of HSC-to-erythroid differentiation. (A) Heatmap generated by Pseudotimecascade visualizing the gene expression cascade. (B) GO enrichment analysis for genes with increasing expression patterns. Count refers to the number of genes showing an increasing pattern that are annotated with a specific GO term. GeneRatio is the proportion of these genes (Count) relative to the total number of genes annotated with the GO term. The top 10 GO terms with the highest GeneRatio are shown. (C) Temporal GO enrichment analysis for genes with increasing expression patterns. The *x* axis represents the gene rankings within each group. For example, the first column shows the GO enrichment for the 46 genes with the earliest switch points. The union of the top 5 GO terms with the highest GeneRatio across groups, retaining only those with *q* value <0.05, is shown.

We applied Pseudotimecascade (Fig. 1) to study the differentiation of hematopoietic stem and progenitor cells (HSCs) into the erythroid lineage in human bone marrow [16] (Fig. 2A). The analysis was conducted on a single sample. Genes were grouped and ordered according to their switch points, defined as zero-crossings of scaled and smoothed gene expression values along pseudotime. The gene expression landscape revealed a cascade of regulatory transitions: Early hematopoietic genes (*CEBPA*, *SPI1*, and *CD34*) decreased along pseudotime, while erythroid regulators (*GATA1* and *KLF1*) were activated at intermediate stages, followed by terminal differentiation genes (*HBA1* and *HBA2*) in late pseudotime. The temporal ordering highlights a regulatory hierarchy in erythropoiesis, in which *GATA1* activation precedes its downstream targets, including *HBB*, *HBA1*, and *HBA2* [17], consistent with its role as a master regulator. Supporting evidence shows that *GATA1* depletion reduces globin gene expression required for erythroid maturation [18–20]. In addition, *TAL1* co-activation supports early lineage specification [21], further reinforcing the coordinated regulatory program underlying erythropoiesis. In contrast, *GATA2*, an antagonist of *GATA1* in the GATA switch, was progressively repressed, reinforcing terminal commitment [22] (Fig. 2B).

Zero-crossing, the default option in Pseudotimecascade for identifying switch points, provides a straightforward interpretation of when gene expression transitions between high and low values. In addition to zero-crossing, Pseudotimecascade supports alternative switch-point definitions, including threshold-crossing and the maximum first derivative. Threshold-crossing is conceptually similar to zero-crossing but allows the use of a user-defined gene expression threshold that differs from zero. The maximum first derivative criterion identifies the pseudotime at which gene expression changes most rapidly, corresponding to the point of strongest transcriptional activation or repression. When applied to the same dataset, both the threshold-crossing and maximum first derivative approaches produce gene grouping and ordering results that are highly consistent with those obtained using zero-crossing (Fig. S1). Gene ordering inferred by Pseudotimecascade is robust to variations in model fitting parameters (Fig. S2A and B) and to perturbations applied to pseudotime values (Fig. S3). Beyond visualizing temporal patterns of individual genes, Pseudotimecascade also enables visualization of pathway activities, calculated using methods such as single-sample gene set enrichment analysis (ssGSEA) [23] (Fig. S4).

**Fig. 3.**
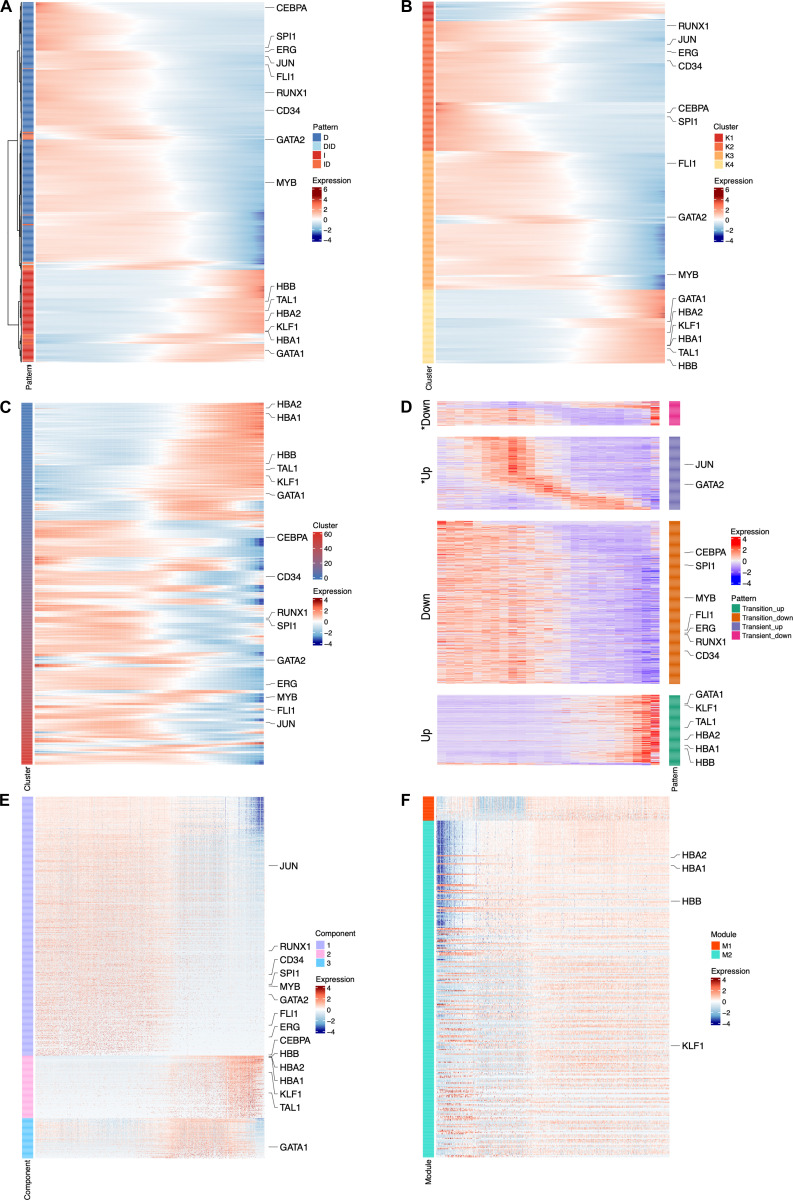
Comparison of gene ordering produced by competing methods. (A to F) Results for hierarchical clustering (A), *k*-means clustering (B), tradeSeq (C), ImpulseDE2 (D), NMF (E), and WGCNA (F).

We further compared Pseudotimecascade with 2 categories of competing methods (Fig. 3). The first category includes approaches that operate on the same scaled and smoothed gene expression trajectories used by Pseudotimecascade, but rely on hierarchical clustering or *k*-means clustering to group and order genes, thereby providing a direct comparison to the gene grouping and ordering module of Pseudotimecascade. The second category consists of standalone methods with their own procedures for gene expression smoothing, grouping, or ordering, including tradeSeq [24], ImpulseDE2 [25], NMF [26], and weighted gene co-expression network analysis (WGCNA) [27]. Most competing methods recover broad global trends in gene expression dynamics that are consistent with Pseudotimecascade, such as the gradual down-regulation of early hematopoietic genes and the up-regulation of erythroid-specific genes along pseudotime. However, none of these methods resolves the fine-grained temporal cascade of gene expression underlying HSPC differentiation captured by Pseudotimecascade, such as the precise temporal ordering of GATA1, HBB, HBA1, and HBA2. A key reason is that most methods do not provide a mechanism to order genes according to their temporal transition points. While ImpulseDE2 reveals a coarse temporal progression of genes, its temporal resolution is lower than that of Pseudotimecascade, and the ordering of transitions among key genes cannot be clearly discerned from the visualization. This limitation arises because ImpulseDE2 was originally designed for bulk RNA-seq data with replicates rather than for scRNA-seq and pseudotime analysis, and therefore cannot fully leverage the temporal resolution afforded by single-cell data. Together, these results highlight the unique ability of Pseudotimecascade to resolve fine-scale temporal gene expression cascades beyond global trends, providing enhanced biological interpretability in pseudotime analysis.

Temporal GO analysis is another unique feature of Pseudotimecascade that identifies enriched pathways at different stages of a cell trajectory. As shown in Fig. 2C, GO terms enriched among genes with increasing expression patterns align with the biology of HSPC differentiation. Early-switch genes are enriched for cell cycle and remodeling processes, such as chromosome segregation and sister chromatid separation, reflecting the proliferative and dynamic nature of progenitors. In contrast, late-switch genes are enriched for erythroid-specific processes, including hemoglobin biosynthesis, oxygen transport, and erythrocyte development, consistent with terminal erythroid commitment. This temporal separation of GO terms highlights the ability of Pseudotimecascade to capture stage-specific regulatory programs. The ranking of GO terms remains highly consistent across different bin sizes and step sizes (Fig. S2C).

**Fig. 4.**
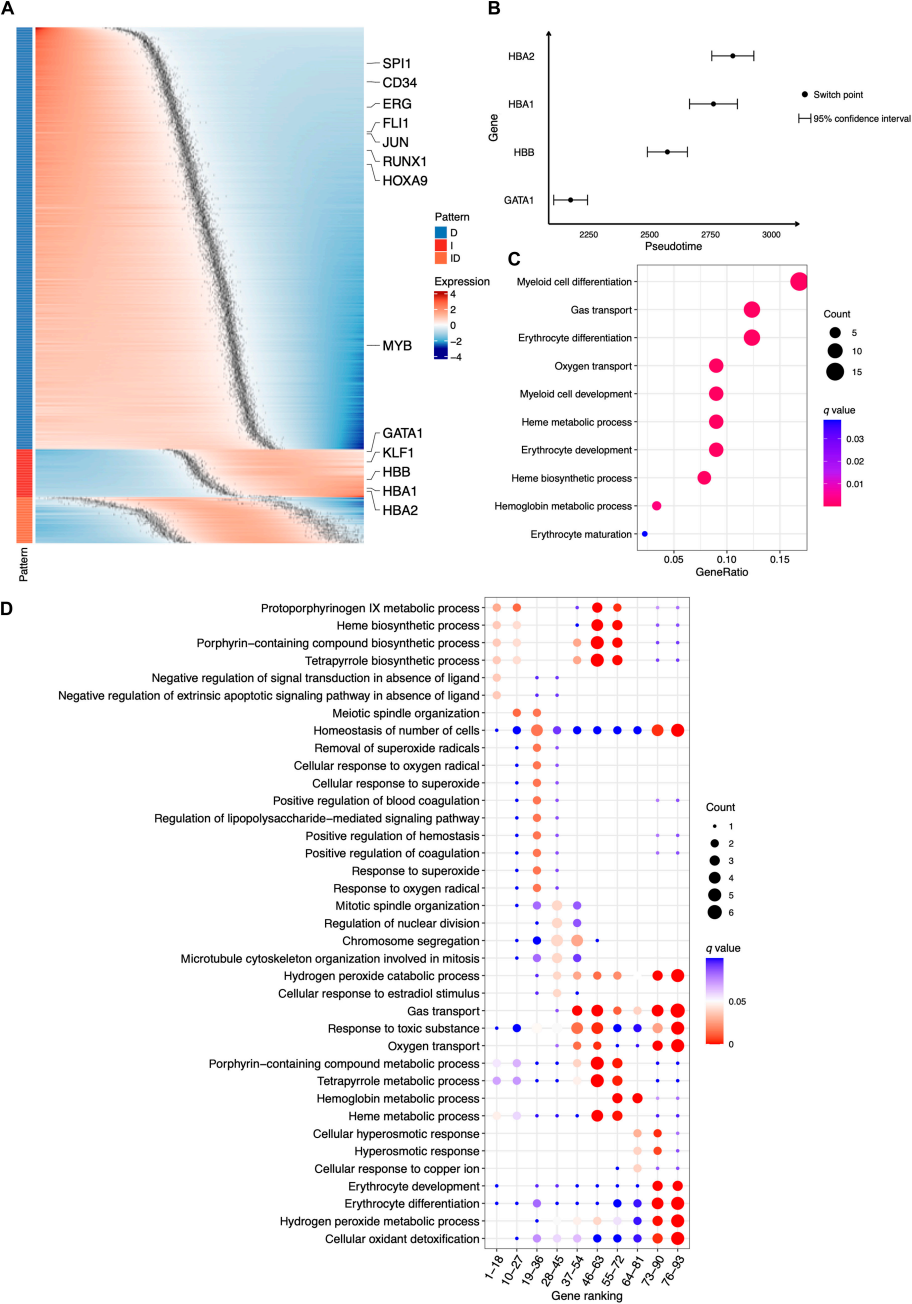
Multi-sample Pseudotimecascade analysis of HSC-to-erythroid differentiation. (A) Heatmap of fitted gene expression trajectories ordered by Pseudotimecascade along the inferred pseudotime axis. (B) Comparison of gene-specific switch points with confidence intervals across 4 genes. (C) GO enrichment analysis for genes with increasing expression patterns. (D) Temporal GO enrichment analysis for genes with increasing expression patterns.

We also applied Pseudotimecascade to analyze HSC differentiation across multiple bone marrow samples (Fig. 4A). Sample integration was performed prior to pseudotime trajectory construction to ensure that pseudotime values are comparable across samples. We observed highly consistent patterns relative to the single-sample analysis, including a progressive decrease in the expression of early hematopoietic genes such as SPI1 and CD34 along pseudotime, activation of erythroid regulators GATA1 and KLF1 at intermediate stages, and induction of terminal differentiation genes HBA1 and HBA2 at later stages. By leveraging switch-point information from multiple samples, Pseudotimecascade can construct confidence intervals for gene-specific switch points and statistically compare the temporal ordering of switch points across genes. For example, the upper bound of the confidence interval for the GATA1 switch point occurs earlier than the lower bounds of the confidence intervals for HBB, HBA1, and HBA2 (Fig. 4B), indicating that the switch point of GATA1 is significantly earlier than those of HBB, HBA1, and HBA2. This finding both confirms and extends our observations from the single-sample analysis. The multi-sample analysis identified GO terms consistent with those detected in the single-sample analysis, with strong enrichment of erythrocyte differentiation-related processes among genes exhibiting monotonically increasing expression patterns along pseudotime (Fig. 4C). The temporal GO analysis likewise recapitulated the single-sample findings (Fig. 4D). Early-switch genes in the multi-sample analysis were enriched for cell cycle-associated processes, including mitotic chromosome dynamics such as chromosome segregation, reflecting the highly proliferative state of progenitor cells. In contrast, late-switch genes showed strong and reproducible enrichment for erythroid-specific processes across samples, including hemoglobin metabolic and biosynthetic processes, oxygen and gas transport, and erythrocyte development and differentiation. These pathways mirror those identified in the single-sample analysis and are characteristic of terminal erythroid commitment. Taken together, the concordance between single-sample and multi-sample analyses in both early and late temporal programs supports the robustness of the inferred pseudotime-resolved regulatory structure across samples.

**Fig. 5.**
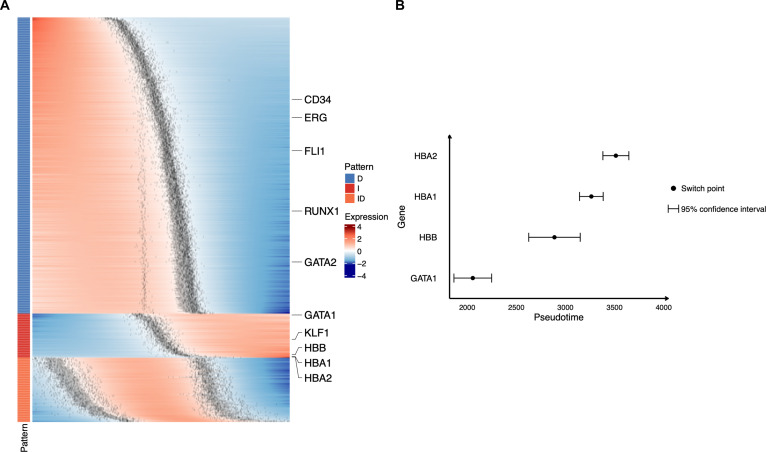
Multi-sample Pseudotimecascade analysis of HSC-to-erythroid differentiation in a second dataset. (A) Heatmap of fitted gene expression trajectories ordered by Pseudotimecascade along the inferred pseudotime axis. (B) Comparison of gene-specific switch points with confidence intervals across 4 genes.

Finally, we applied Pseudotimecascade to a second scRNA-seq dataset from human bone marrow [28], which contains cells from 12 donors. Using the same multi-sample pipeline, we observed highly consistent patterns compared with the multi-sample analysis of the first dataset, including a progressive decrease in the expression of early hematopoietic genes such as CD34 along pseudotime, activation of erythroid regulators GATA1 and KLF1 at intermediate stages, and induction of terminal differentiation genes HBA1 and HBA2 at later stages (Fig. 5A). Moreover, the upper bound of the confidence interval for the GATA1 switch point occurs earlier than the lower bounds of the confidence intervals for HBB, HBA1, and HBA2 (Fig. 5B), indicating that the switch point of GATA1 is significantly earlier than those of HBB, HBA1, and HBA2, consistent with the pattern observed in the first dataset.

## Conclusion

In conclusion, Pseudotimecascade provides a unified and scalable framework for dissecting coordinated gene expression programs along pseudotime trajectories. By moving beyond single-gene analysis to jointly model temporal switch points and relative gene ordering, it enables the identification of fine-scale regulatory cascades and hierarchical relationships that are not readily resolved by existing approaches. The incorporation of temporal GO enrichment further links dynamic transcriptional patterns to stage-specific biological functions, enhancing interpretability. Across multiple scRNA-seq datasets of hematopoietic differentiation, Pseudotimecascade demonstrates robustness, reproducibility, and biological coherence, highlighting its value as a complementary tool for elucidating complex gene expression dynamics in continuous cellular processes.

## Materials and Methods

### Pseudotimecascade

#### Input

Pseudotimecascade takes as input a numerical matrix with *N* cells and a corresponding vector specifying the cell ordering. The matrix may represent a scRNA-seq gene expression matrix or a matrix of pathway-level activity scores derived using methods such as ssGSEA [23]. Proper preprocessing, such as data normalization, should be performed on the gene expression matrix beforehand. By default, a gene is retained if its expression exceeded 0.1 in more than 5% of cells. The cell ordering can be obtained from pseudotime or RNA velocity methods or, more generally, from any biologically meaningful approach. Pseudotimecascade reorders the columns of the gene expression matrix so that the column names align with the specified cell ordering. By default, the pseudotime value of the *i*th cell is assigned as *i*. Alternatively, users may provide a custom vector of pseudotime values as input.

Pseudotimecascade supports scRNA-seq analysis from either a single sample or multiple samples. When multiple samples are analyzed, sample integration and batch correction [29,30] are required prior to pseudotime trajectory construction to mitigate systematic differences across samples. Importantly, this integration step is not performed directly on the input gene expression matrix.

#### Fitting temporal gene expression patterns

For each gene *j*, Pseudotimecascade fits a smoothed curve to its expression values and tests whether the gene exhibits significant variation along pseudotime. Specifically, Pseudotimecascade models gene expression as a smooth function of pseudotime using a GAM within the VGAM framework [31]. For each gene, a Tobit regression model is fitted to explicitly account for left-censoring at low expression values, using the tobit family with a default lower censoring threshold of 0.1 and the mean.obs fitted-value parameterization. The model is written as $y=f\left(\mathrm{pt}\right)+\varepsilon$, where $f\left(\cdot \right)$ denotes a smooth function of pseudotime and ε follows a censored Gaussian distribution. Trajectory nonlinearity is accommodated by modeling $f\left(\mathrm{pt}\right)$ using a spline basis specified as $s\left(\mathrm{pt},\;\mathrm{df}=2\right)$ by default, which captures nonlinear temporal trends while maintaining low model complexity. The degrees of freedom of the spline are constant rather than optimized from the data, providing explicit regularization to prevent overfitting and ensuring that only broad, biologically interpretable temporal patterns are captured. Model fitting is performed using the vgam function with a maximum of 50 iterations by default to ensure numerical stability and convergence. *P* values are adjusted for multiple testing using the Benjamini–Hochberg (BH) procedure [32], and only genes with adjusted *P* values <0.05 are retained for downstream analysis. The fitted gene expression values are further scaled to have a mean of 0 and a standard deviation of 1.

If the input data contain multiple samples, the above fitting procedure is performed separately within each sample.

#### Identifying switch points

Pseudotimecascade supports 3 methods for identifying switch points. By default, switch points are defined as zero-crossings of the scaled and smoothed gene expression trajectory along pseudotime. Specifically, let $x_{\mathrm{ij}}$ denote the scaled and smoothed expression value of gene *j* in cell *i*. Pseudotimecascade identifies a set of switch points $S_{j}$, defined as the indices of cells at which the fitted expression trajectory crosses 0, $\begin{array}{c} S_{j}=\left\{\;i\in \left\{1,\ldots ,\;N-1\right\}:x_{\mathrm{ij}}x_{i+1,j}<\;0\;\right\}. \end{array}$ Similarly, switch points can be defined using a threshold-crossing criterion applied to the scaled and smoothed gene expression trajectory along pseudotime. Specifically, $\begin{array}{c} S_{j}=\left\{\;i\in \left\{1,\ldots ,\;N-1\right\}:\left(x_{\mathrm{ij}}-\gamma \right)\left(x_{i+1,j}-\gamma \right)<0\;\right\} \end{array}$, where γ is a user-specified constant that defines the gene expression threshold. Finally, Pseudotimecascade can also identify the switch point as the location corresponding to the maximum absolute first derivative of the temporal gene expression trajectory, defined as $S_{j}=\arg\;\max_{i\in \left\{1,\ldots ,N-1\right\}}\left|\frac{\partial x_{\mathrm{ij}}}{\partial t_{i}}\right|$, where $t_{i}$ denotes the pseudotime of cell *i*, and the first derivative is approximated as $\frac{\partial x_{\mathrm{ij}}}{\partial t_{i}}=\frac{x_{i+1,j}-x_{i,j}}{t_{i+1}-t_{i}}$.

When the input data contain multiple samples, switch points are identified separately within each sample. For each gene, a temporal-pattern label and an adjusted *P* value are computed per sample. A gene is considered valid in a sample if its adjusted *P* value is smaller than 0.05. An overall temporal pattern is then defined as the most frequently observed pattern among valid samples. Cross-sample agreement is quantified using a confidence score, defined as the proportion of samples supporting this overall pattern. By default, genes are retained if the confidence score is at least 0.75, allowing up to 25% of samples to deviate from the dominant pattern. For retained genes, only samples supporting the overall pattern are used in downstream aggregation. A mean fitted trajectory is computed by averaging fitted expression values across supporting samples at each pseudotime grid point. Switch points are subsequently re-identified from this mean trajectory using the same definition as in the single-sample setting. For each switch point, cross-sample variability is summarized using switch-point positions estimated separately in each supporting sample, and a confidence interval is reported as mean ± 2 SE (standard error) across samples.

#### Gene grouping and ordering

Only genes that pass the significance threshold in the single-sample setting or are retained in the multi-sample setting are included in downstream analyses and subsequently assigned temporal expression patterns. Pseudotimecascade classifies each gene according to the temporal pattern of its fitted trajectory. The temporal pattern is encoded by the local direction at each switch point, denoted as I (increasing) if $f_{j}<f_{j+1}$ and D (decreasing) otherwise; directions are concatenated when multiple switch points are present. Based on this encoding, genes are classified as monotonically increasing, monotonically decreasing, or nonmonotonic (e.g., increasing–decreasing and decreasing–increasing).

Pseudotimecascade then groups genes according to their temporal patterns, which are ordered by increasing complexity (i.e., monotonic patterns followed by nonmonotonic patterns). Within each temporal pattern, genes are further ordered by the location of their first switch point in ascending order. For visualization, the top 1,000 genes ranked by adjusted *P* values are selected to enhance interpretability while preserving the overall structure of temporal expression patterns. The scaled fitted expression values of the ordered genes are visualized in a heatmap, with an option to highlight the names of key marker genes. Optionally, switch-point locations can be displayed on the heatmap as overlaid point markers, without affecting gene ordering or temporal-pattern assignment.

#### GO enrichment analysis

Pseudotimecascade performs 2 types of GO enrichment analyses within each temporal pattern. In the first type of analysis, enriched GO terms are identified across all genes with a temporal pattern. In the second type of analysis, Pseudotimecascade performs temporal GO analysis by grouping genes with similar switch points using a sliding-window approach and conducting enrichment analysis within each group. Specifically, the sliding-window approach partitions the ordered gene list within a temporal pattern into overlapping bins, where each bin contains 20% of the genes and is shifted by 10% along the ranked list. This results in approximately 10 partially overlapping gene groups, each of which is subjected to GO enrichment analysis to characterize functional changes along pseudotime. Since gene sets used for functional enrichment analysis are recommended to be restricted to approximately 20 to 200 genes to achieve robust and biologically interpretable results [33], this strategy balances the size of individual bins with the total number of bins, ensuring both sufficient gene counts for enrichment analysis and adequate resolution along pseudotime. The bin size and step size are user configurable parameters, with default values set to 20% and 10%, respectively.

### Data collection and processing

The first scRNA-seq dataset from human bone marrow was downloaded from the Human Cell Atlas [16] and processed using the Seurat R package. Cells were filtered to retain high-quality transcriptomes by restricting to 400 to 4,000 detected genes per cell and a mitochondrial RNA percentage ≤10%. After quality control, gene count matrices were normalized using log-normalization with a scale factor of 10,000. Highly variable genes were identified using the variance-stabilizing transformation (VST) method with the top 2,000 features selected, and expression values were standardized across these genes. Dimensionality reduction was performed using principal components analysis (PCA), followed by Uniform Manifold Approximation and Projection )UMAP) embedding based on the first 30 principal components. A shared nearest-neighbor graph was constructed using the same principal components, and Louvain clustering was performed at a resolution of 1.0. Cell types were annotated using canonical lineage marker genes, with the HSC population defined by expression of stem-like markers including CD34, KIT, SOX4, and SPINK2, and erythroid-lineage cells identified based on expression of erythroid markers including HBD and GYPA. To model the differentiation trajectory from HSCs toward the erythroid lineage, subsequent analyses were restricted to these 2 annotated populations, which were used to define the start and end states of a continuous developmental trajectory. Pseudotime ordering was inferred using the TSCAN framework, where exprmclust was applied to the UMAP embedding to construct a trajectory model and TSCANorder was used to derive an ordered cell sequence. To enable pathway-level temporal analysis along pseudotime, we applied ssGSEA [34] to compute pathway-activity scores at the single-cell level for gene sets corresponding to GO Biological Process (BP) terms containing at least 10 genes.

The second scRNA-seq dataset from human bone marrow [28] was obtained from the Gene Expression Omnibus (GEO) under accession number GSE253355. We directly downloaded the processed and annotated Seurat object without reprocessing the raw sequencing data. The analysis was restricted to cell populations corresponding to the established erythroid developmental hierarchy. Specifically, cells annotated as HSCs, megakaryocyte–erythroid progenitors (MEPs), erythroblasts, and late-erythroid cells were selected based on the provided cell-type annotations. Pseudotime ordering was inferred using the TSCAN framework. The low-dimensional embedding available in the processed Seurat object was used as input for trajectory construction, together with the selected cluster annotations to guide lineage ordering. Cells were ordered along a single continuous trajectory spanning HSC to late-erythroid states.

### Competing methods

A set of competing methods was applied to generate alternative gene orderings. To facilitate comparison with Pseudotimecascade, the same set of genes visualized by Pseudotimecascade was displayed in heatmaps for each competing method.

#### Hierarchical clustering

Hierarchical clustering was applied to the scaled and fitted gene expression values of the same set of genes visualized by Pseudotimecascade. Specifically, Euclidean distance was used to quantify pairwise similarity between genes, and hierarchical clustering was performed using Ward’s linkage, ward.D2, with the resulting dendrogram determining the row order of genes in the heatmap.

#### *K*-means clustering

*K*-means clustering was applied to the scaled and fitted gene expression values of the same set of genes visualized by Pseudotimecascade. Specifically, Euclidean distance was used to quantify pairwise similarity between genes, and *k*-means clustering was performed with *k* = 4 clusters and 50 random initializations. The gene clusters were first ordered in increasing order of cluster identity, that is, cluster 1, cluster 2, cluster 3, and cluster 4. Within each cluster, genes were ordered by ascending adjusted *P* values from the GAM model.

#### tradeSeq

The same pseudotime values and scRNA-seq gene expression matrix used as input to Pseudotimecascade were also used as input to tradeSeq (version 1.18.0). For each gene, smooth expression trajectories along pseudotime were fitted using the tradeSeq function fitGAM, which models gene expression using a negative binomial GAM (NB-GAM). The fitted trajectories were evaluated on a common pseudotime grid and standardized by gene-wise *z*-score scaling to emphasize temporal expression patterns. Genes were first grouped using the tradeSeq function clusterExpressionPatterns, which produces a collection of fine-grained expression pattern clusters. As tradeSeq does not provide an intrinsic gene ranking scheme, clusters were first ordered according to the numeric labels returned by clusterExpressionPatterns, and genes within each cluster were then ordered by ascending adjusted *P* values from the fitGAM function.

#### ImpulseDE2

To apply ImpulseDE2 to single-cell pseudotime data, cells were first ordered according to TSCAN-inferred pseudotime, and the continuous trajectory was discretized into 25 quantile-based time bins. Within each bin, cells were randomly partitioned to generate 3 pseudobulk replicates by summing gene-level counts, yielding a time-series count matrix with repeated samples per time point. This binning and pseudobulk construction were introduced to adapt single-cell pseudotime data to the bulk time-course input structure assumed by ImpulseDE2, which requires sample-level counts at discrete time points with replicate-level variability. Gene-wise temporal models were then fitted using runImpulseDE2 with boolCaseCtrl = FALSE and boolIdentifyTransients = TRUE, enabling likelihood-based testing of temporal differential expression and identification of transient and monotonic expression patterns. Genes were considered significantly dynamic based on adjusted *P* values, *P*_adj_ ≤ 0.05 together with model-convergence and expression-quality criteria. Expression heatmaps were generated using the ImpulseDE2 function plotHeatmap, which classifies genes into transition and transient categories with up-regulated or down-regulated trajectories and determines gene ordering within each category based on features of the fitted temporal profiles. The default ImpulseDE2 grouping and ordering were retained for visualization.

#### Matrix factorization-based method (NMF)

NMF was used as a matrix factorization-based baseline method to identify gene components directly from the log-normalized gene-by-cell expression matrix. Unlike pseudotime-aware models, NMF does not explicitly model temporal ordering and instead decomposes the expression matrix into latent components under nonnegativity constraints. As required by NMF, genes with zero expression across all cells were removed prior to analysis. NMF was performed using the R function nmf::nmf [26] with default settings to assign genes to components. Because NMF does not provide an intrinsic ordering among components, components were first ordered according to their numeric labels, for example, 1, 2, and 3. Within each component, genes were then ordered by their contribution to the assigned component, defined as the magnitude of their loadings in the basis matrix. For visualization, expression values were standardized using row-wise *z* scores, and cells were ordered by pseudotime.

#### Weighted gene co-expression network analysis

WGCNA is primarily designed to identify and analyze co-expression structures at the module level rather than to compare individual genes. The pseudotime-ordered, log-normalized cell-by-gene matrix was used as input for WGCNA following the standard WGCNA tutorial. Gene co-expression modules were constructed using the blockwiseModules function in the WGCNA package with default parameters. The soft-thresholding power was selected using pickSoftThreshold over a candidate range of 1 to 20; when an optimal value could not be determined automatically, a default value of β=6 was adopted. In the blockwiseModules function, modules were identified based on hierarchical clustering of topological overlap matrices combined with dynamic tree cutting. Genes assigned to the gray module, which represents unassigned genes that do not belong to any coherent co-expression module in WGCNA, were excluded from downstream analyses. In total, 140 non-gray co-expression modules were identified. To characterize module-level dynamics along pseudotime, module eigengenes (MEs) computed by WGCNA were correlated with pseudotime values, and modules were ordered according to the strength of this correlation. While WGCNA does not intrinsically impose an ordering of genes within modules, gene-level ordering was obtained using a customized procedure. Specifically, signed module membership (kME) values were calculated using the signedKME function, as recommended by the WGCNA framework, which measures the correlation between each gene’s expression profile and the eigengene of its assigned module. Genes within each module were then ordered by the absolute value of their kME such that genes most representative of each module appeared first. For visualization, gene expression values were standardized using row-wise *z* scores.

## Data Availability

Pseudotimecascade (version 1.0) is freely available at https://github.com/changxinw/Pseudotimecascade. The software requires R (version ≥3.5.0) and the following R packages: ComplexHeatmap, VGAM, circlize, dplyr, ggplot2, clusterProfiler, org.Hs.eg.db, and org.Mm.eg.db. An example dataset is included in the package to facilitate testing and reproducibility.
