# Supplementary material for: Pseudotimecascade Visualizes Gene Expression Cascade in Pseudotime Analysis
Source: Comput Struct Biotechnol J. 2026 Mar 6;35(1):0007. doi: 10.34133/csbj.0007 (PMC13047748; doi:10.34133/csbj.0007)
Supplement: Supplementary 1 — Figs. S1 to S4 [file csbj.0007.f1.pdf]

Supplementary materials

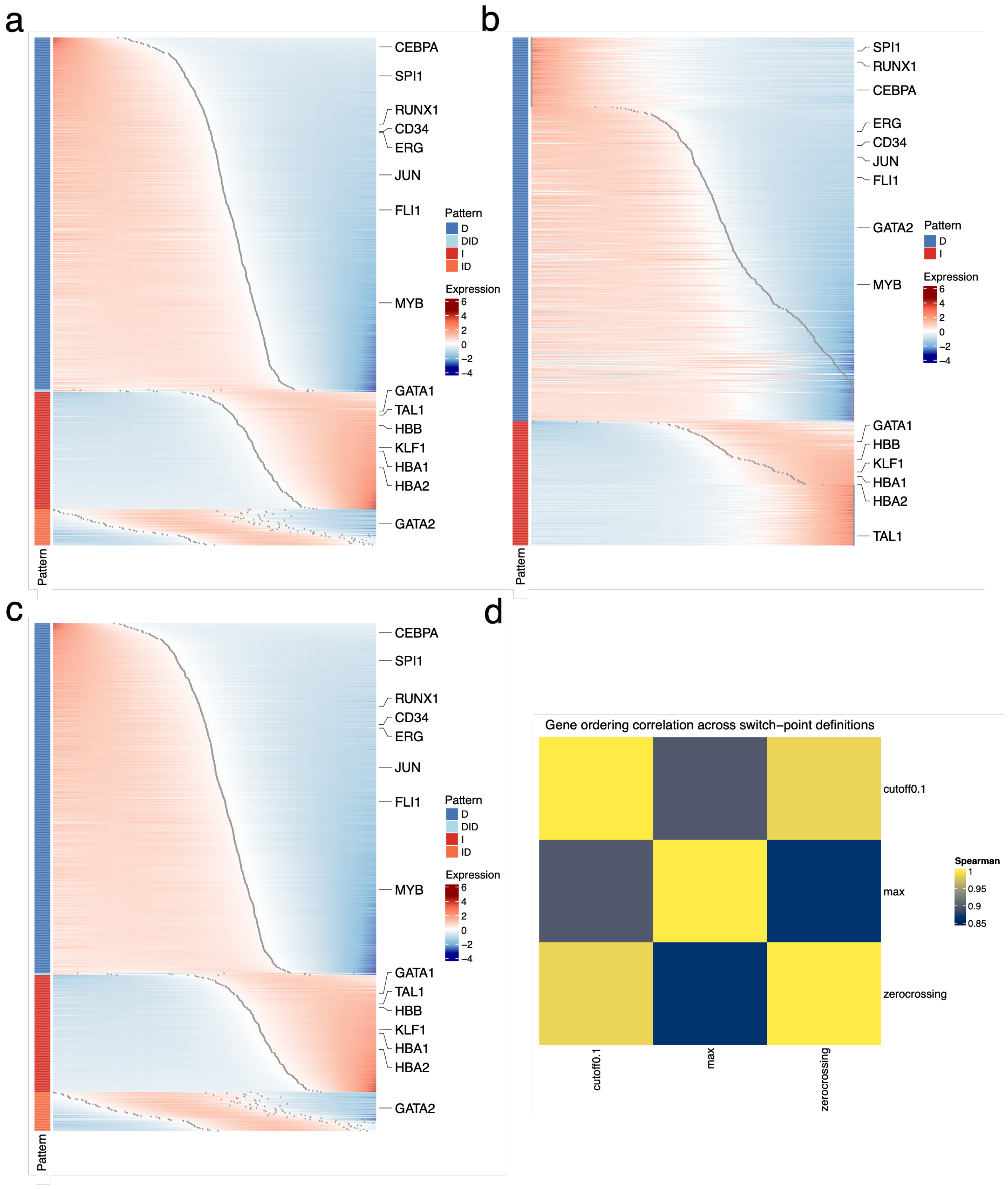

Figure S1. Robustness of gene ordering to alternative switch-point definitions. a–c, Heatmaps of scaled fitted gene expression trajectories ordered by Pseudotimecascade using zero-crossing (**a**), maximum first derivative (**b**), and threshold-crossing with cutoff = 0.1 (**c**). In all cases, genes were first filtered by significance and restricted to the top 1,000 genes ranked by adjusted *p*-values. Only genes assigned to the same temporal pattern across methods were retained for ordering comparison. **d**, Pairwise Spearman correlations of gene ordering across switch-point definitions, computed using the ordering of pattern-consistent genes.

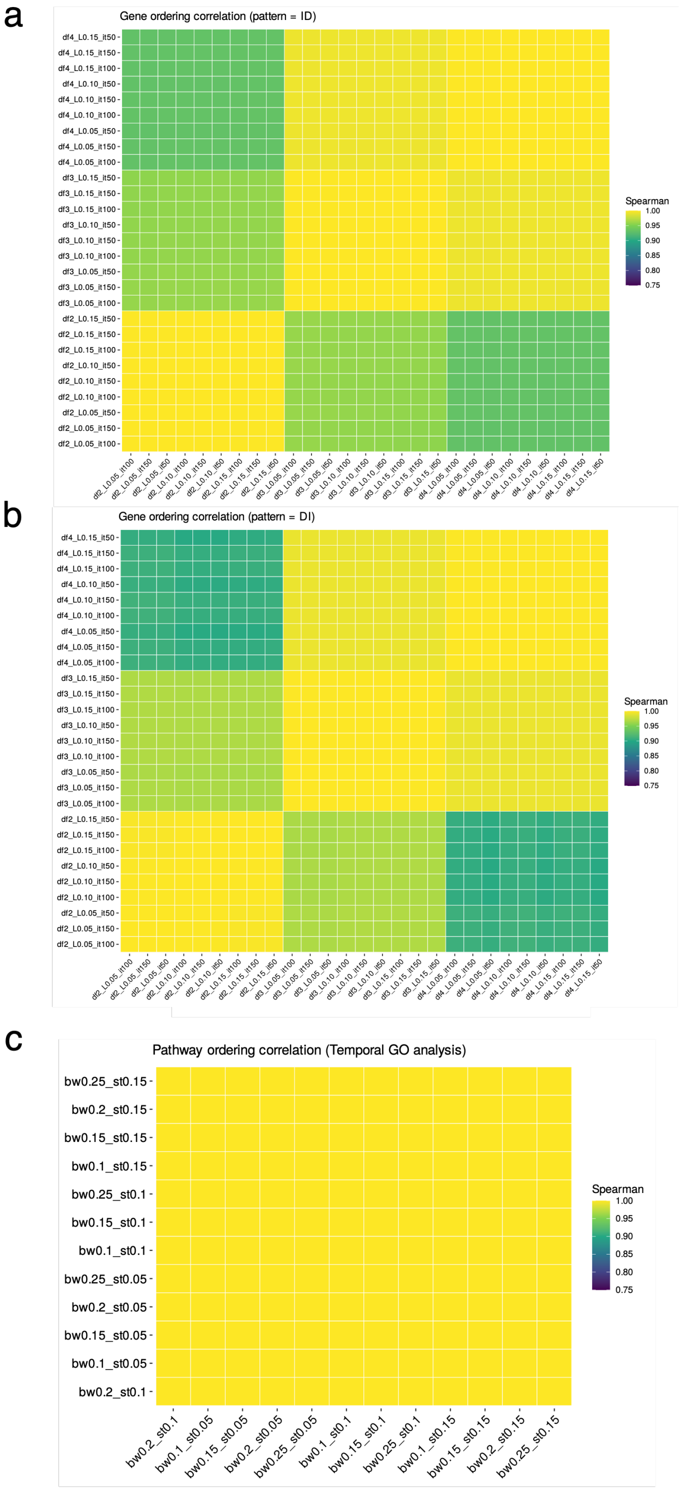

Figure S2. a–b, Robustness of gene ordering to variations in model fitting parameters. For each parameter setting, genes were first filtered by significance and restricted to the top 1,000 genes ranked by adjusted p-values. Only genes assigned to the same temporal pattern under all compared settings were retained. Pairwise Spearman correlations were then computed based on the ordering of switch points within each pattern. **a**, Gene ordering correlations for genes assigned to the increasing–decreasing (ID) pattern. **b**, Gene ordering correlations for genes assigned to the decreasing–increasing (DI) pattern. **c**, Robustness of pathway ordering in temporal GO analysis to sliding-window parameter choices. Temporal GO enrichment was performed using different combinations of bin width and stride. For each setting, significantly enriched pathways (adjusted $p \leq 0.05$) were identified within each bin, and the top five pathways per bin were retained for visualization. Only pathways also detected under the default parameter setting were kept, and pairwise Spearman correlations of pathway rank ordering were computed across parameter settings.

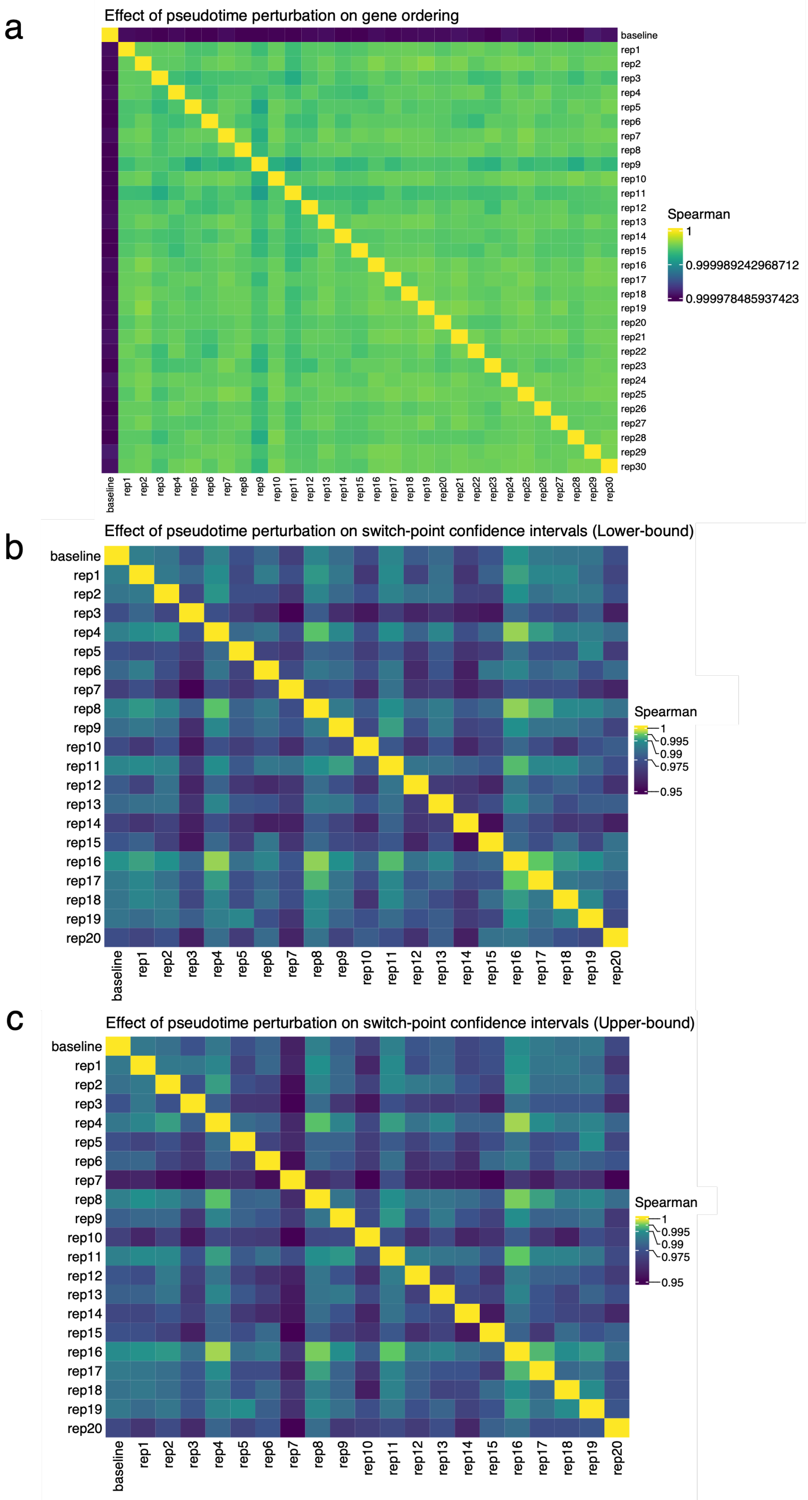

Figure S3. Robustness of gene ordering and switch-point confidence intervals to pseudotime perturbation. a, Robustness of gene ordering in the single-sample analysis under pseudotime perturbation. Gaussian noise (mean=0, sd=0.5) was added to pseudotime values, and gene ordering was recomputed. Genes were first filtered by significance and restricted to those with consistent temporal pattern assignments before and after perturbation. Pairwise Spearman correlations were then computed between gene orderings obtained from the original and perturbed pseudotime values. **b–c**, Robustness of switch-point confidence intervals in the multi-sample analysis under pseudotime perturbation. For each perturbation, switch points were re-estimated within each sample, and confidence intervals were constructed across samples. Pairwise Spearman correlations were computed between perturbations based on the lower bounds (**b**) and upper bounds (**c**) of switch-point confidence intervals, respectively.

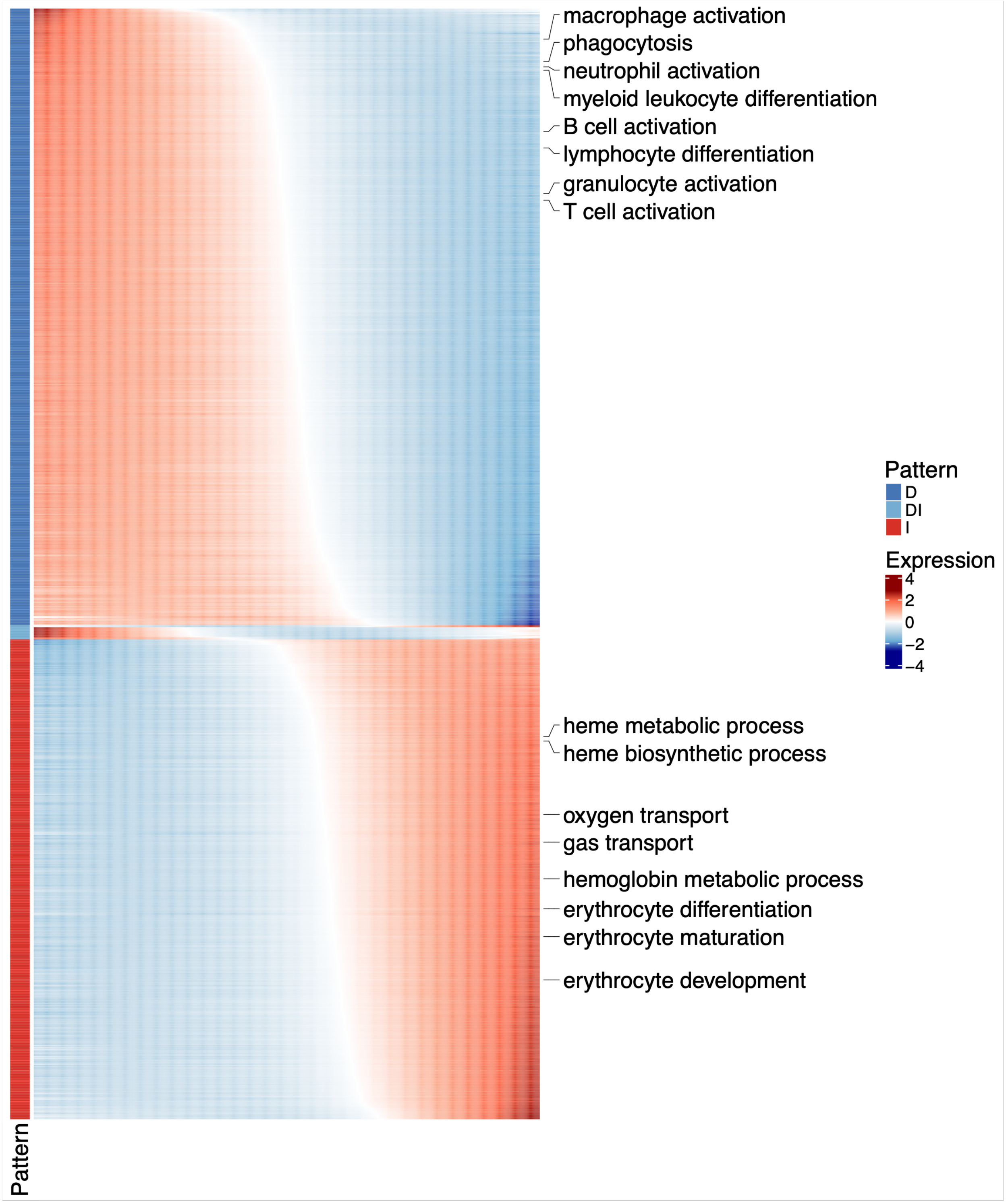

Figure S4. Pathway activity dynamics along pseudotime inferred by ssGSEA. Single-sample gene set enrichment analysis (ssGSEA) was used to compute pathway activity scores for Gene Ontology (GO) biological process terms at the single-cell level. Pathways were first filtered by significance and restricted to the top 1,000 pathways ranked by adjusted *p*-values. Pathway activity profiles were then ordered according to TSCAN-inferred pseudotime and visualized as heatmaps.
